# Unveiling the Unprecedented: An Astonishing Rarity of Metoclopramide Hydrochloride-Triggered Nystagmus in a Pregnant Woman

**DOI:** 10.7759/cureus.40842

**Published:** 2023-06-23

**Authors:** Dharmesh J Patel, Kamlesh Chaudhari, Deepti Shrivastava, Apoorva Dave, Akruti Shinde, Harshith Gowda

**Affiliations:** 1 Department of Obstetrics and Gynaecology, Jawaharlal Nehru Medical College, Datta Meghe Institute of Higher Education and Research, Wardha, IND; 2 Department of Obstretics and Gynaecology, Jawaharlal Nehru Medical College, Datta Meghe Institute of Higher Education and Research, Wardha, IND; 3 Department of Radiology, Jawaharlal Nehru Medical College, Datta Meghe Institute of Higher Education and Research, Wardha, IND

**Keywords:** vomiting, nausea, nystagmus, extrapyramidal side effects, metoclopramide hydrochloride

## Abstract

Metoclopramide hydrochloride is a widely used medication for the treatment of gastrointestinal disorders such as nausea, vomiting, and gastroparesis. However, it has been associated with extrapyramidal side effects (EPS) such as tardive dyskinesia, nystagmus, and other locomotive disorders on rare occasions. These reactions are commonly seen in children and females, particularly in young people. In this article, we report a rare case of a 15-week pregnant woman who was prescribed metoclopramide hydrochloride in view of nausea and vomiting, which was later diagnosed as vomiting in pregnancy not relieved with first-line medications, and has later developed drug-induced nystagmus, highlighting its unpredictable nature and shortcomings of management in the pregnant woman. This article will draw the attention of obstetricians and gynecologists to wisely prescribe metoclopramide hydrochloride for treating nausea and vomiting in pregnant women.

## Introduction

Metoclopramide hydrochloride has been widely used for treating nausea, vomiting, gastroparesis, gastroesophageal reflux syndrome, and migraines. It is a well-known antiemetic and prokinetic agent that works as a selective dopamine receptor (D2) antagonist, thus blocking the action of dopamine, a neurotransmitter, in the gut and chemoreceptor trigger zone [1]. However, its use has been associated with the development of extrapyramidal side effects (EPS) with an incidence of approximately 0.2% [2]. These side effects are involuntary movements or muscle spasms caused by their effects on the central nervous system. EPS can include symptoms such as akathisia (restlessness), dystonia (abnormal muscle tone), Parkinsonism (tremor, rigidity, and bradykinesia), and nystagmus. It can be dose-related and more common with long-term use of metoclopramide [3]. Nystagmus is characterized by a repetitive and involuntary eye movement that can occur horizontally, vertically, or rotationally. In some cases, the use of metoclopramide hydrochloride has been reported to cause nystagmus, particularly when it is used in high doses or for extended periods of time [4]. Metoclopramide hydrochloride has been prescribed as second-line therapy for the management of nausea and vomiting in pregnancy due to the risk of EPS [5]. The case described in this report showed one of the rarest adverse reactions to the drug nystagmus.

## Case presentation

A 22-year-old primigravida presented to the emergency room with chief complaints of nausea and vomiting for one month. The patient was 15 weeks pregnant. She had six episodes of vomiting since the morning of presentation to the emergency room. She had a low-grade fever and headache for about one week, which was alleviated with antipyretic. The patient was previously advised to take oral rehydration solution and doxylamine 10 mg tablet for nausea and vomiting as an outpatient in her previous visits, but her symptoms were not relieved. She was then admitted to the obstetrics and gynecology ward in view of positive ketone bodies in the urine and nausea and vomiting not relieved with oral medication.

On admission, the patient’s blood pressure was recorded as 120/70 mmHg, the pulse rate was 100 beats/minute, and the respiration rate was 22 breaths/minute. Her oxygen saturation level was measured at 98% while breathing in normal air. All routine investigations were done. The patient was started on an injection of metoclopramide hydrochloride 2 mg for every six hours and an injection of ondansetron 4 mg for every 12 hours in an intravenous drip for nausea and vomiting. Her total leucocyte count was found to be elevated to 18,600/μL. Hence, the injections of amoxicillin and clavulanic acid were started via the intravenous route. Her urine routine and microscopy examination suggested ketone as +1 and sugar as +1. The sodium level in the blood was 132 mEq/L, and the potassium level was 2.9 mEq/L.

**Figure 1:**
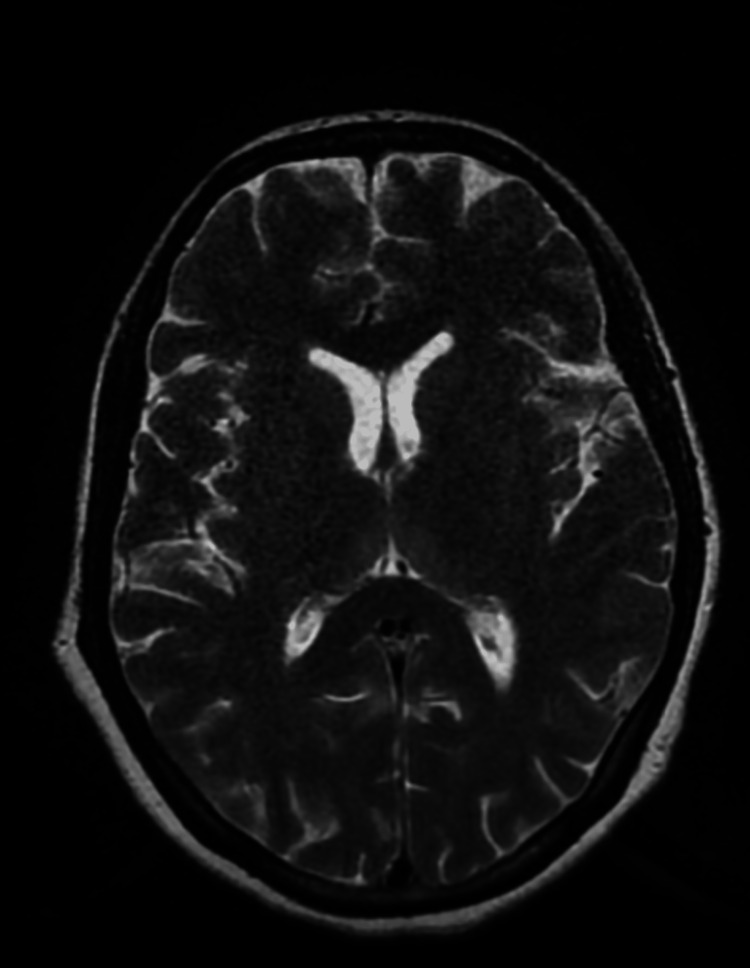
Axial T2WI of plain brain magnetic resonance imaging suggestive of no obvious abnormalities T2WI, T2-weighted image

Other investigations were normal. She was diagnosed with hyperemesis gravidarum and was managed conservatively. Infusion of potassium chloride (KCL) injection 40 mEq in 500 mL Ringer’s lactate drip was intravenously given for hypokalemia. On the third day of the injection of metoclopramide hydrochloride, the patient had an acute convulsive episode with vertical and horizontal nystagmus with debilitating ataxia. Levetiracetam 1 g injection was intravenously given stat. And on an emergency basis, the physician’s opinion was taken. As per the advice of the physician, a brain plain magnetic resonance imaging (MRI) was done, which revealed no significant abnormality (Figure 1). Neurologist opinion was taken in view of this event. Refraction testing, fundus examination, and antinuclear antibody (ANA) test using the immunofluorescence assay (IFA) method were advised by a neurologist. ANA test by IFA suggested a negative result. A complete ophthalmic examination by an ophthalmologist revealed upbeat nystagmus, as shown in Video 1. As per the physician’s advice, the injection of metoclopramide hydrochloride was withheld in suspicion of EPS of the drug. The patient was continued on injection of ondansetron, and the injection of thiamine was started via the intravenous route. The patient’s symptoms reduced significantly after stopping the injection of metoclopramide hydrochloride, and nystagmus also disappeared. The patient was clinically asymptomatic and was discharged on doxylamine 10 mg tablet thrice daily.

**Video 1:** Metoclopramide hydrochloride-induced upbeat nystagmus

## Discussion

Metoclopramide hydrochloride works by blocking both peripherally and centrally located dopamine receptors (D2). Central receptors are located in the medullary chemoreceptor trigger zone in the area postrema. This area is usually activated by substances such as apomorphine or levodopa. The drug also impacts the visceral afferent nerves that transmit information from the gastrointestinal system to the vomiting center in the chemoreceptor trigger zone [6].

Metoclopramide hydrochloride not only inhibits dopamine receptors but also inhibits serotonin type 3 (5HT3) receptors and serotonin type 4 (5HT4) receptors agonists [7]. Metoclopramide hydrochloride counteracts the effects of apomorphine on gastric emptying by enhancing the intensity and length of esophageal contractions. This leads to a heightened state of rest in the lower esophageal sphincter, while at the same time causing the duodenal bulb and pyloric sphincter to relax, which in turn boosts the peristaltic movements in the duodenum and jejunum [8]. Metoclopramide hydrochloride is efficiently absorbed from the gut, with maximum plasma levels reached within one to two hours of oral administration. Its lipid solubility results in a long half-life and widespread distribution, which can vary from 4.5 to 8.8 hours [9].

Metoclopramide hydrochloride is capable of passing through the placenta. If a mother takes metoclopramide hydrochloride during labor, it can cause extrapyramidal symptoms and methemoglobinemia in newborns. The American College of Obstetricians and Gynecologists recommends that metoclopramide hydrochloride should only be used as a last resort for severe nausea and vomiting during pregnancy, with a dose of 5-10 mg every six to eight hours given orally, intramuscularly, or intravenously to adequately hydrated patients. The preferred treatment option is pyridoxine (vitamin B6) or a combination of pyridoxine and doxylamine [10].

The most severe side effects associated with metoclopramide hydrochloride are EPS. Serious neurological events related to metoclopramide hydrochloride can occur relatively soon after treatment begins, usually within the first 24-72 hours or after multiple doses. The likelihood of these events increases with high doses, prolonged treatment, and treatment of children or elderly patients [2]. Tardive dyskinesia and Parkinsonism tend to develop with long-term use, while dystonia and akathisia can happen after just one dose [3]. Other EPS like nystagmus and ataxia can also occur.

Hyperemesis gravidarum is a condition in which persistent vomiting during pregnancy causes weight loss and dehydration, resulting in ketonemia, ketonuria, or both [11]. The condition can have a substantial negative effect on the quality of life of women and their families and may pose a significant challenge in terms of treatment. The incidence of hyperemesis gravidarum ranges from 0.3% to 3%, depending on the literature source [12].

Similarly, in our case, nystagmus was observed in a pregnant woman who was suffering from hyperemesis gravidarum and was prescribed metoclopramide hydrochloride for the same. However, the symptoms resolved after stopping the drug, which suggests to be the EPS of metoclopramide hydrochloride. This suggests that obstetricians and gynecologists should use metoclopramide hydrochloride as the last resource in pregnant women keeping in mind the EPS of it. As with all medications, the use of metoclopramide hydrochloride during pregnancy should be carefully evaluated by a healthcare provider to ensure the best outcome for the mother and her baby.

## Conclusions

In conclusion, this case report highlights the importance of careful evaluation of medication use during pregnancy, particularly with metoclopramide hydrochloride due to its potential for EPS. Obstetricians and gynecologists should reserve its use as a last resort in severe and refractory cases after thorough consideration of the risks and benefits. It is crucial for healthcare providers to closely monitor and weigh the potential outcomes for both the mother and her baby when deciding on the use of metoclopramide hydrochloride during pregnancy.
